# Activation of the integrated stress response in human hair follicles

**DOI:** 10.1371/journal.pone.0303742

**Published:** 2024-06-20

**Authors:** Derek Pye, Rachel Scholey, Sin Ung, Madoc Dawson, Asim Shahmalak, Talveen S. Purba

**Affiliations:** 1 Division Musculoskeletal and Dermatological Sciences, Centre for Dermatology Research, Manchester Academic Health Science Centre, Faculty of Biology, Medicine and Health, School of Biosciences, The University of Manchester, Manchester, United Kingdom; 2 Bioinformatics Core Facility, University of Manchester, Manchester, United Kingdom; 3 Crown Clinic, Manchester, United Kingdom; POSTECH - Pohang University of Science and Technology, REPUBLIC OF KOREA

## Abstract

Unravelling how energy metabolism and stress responses are regulated in human scalp hair follicles could reveal novel insights into the controls of hair growth and provide new targets to manage hair loss disorders. The Mitochondrial Pyruvate Carrier (MPC) imports pyruvate, produced via glycolysis, into the mitochondria, fuelling the TCA cycle. Previous work has shown that MPC inhibition promotes lactate generation, which activates murine epithelial hair follicle stem cells (eHFSCs). However, by pharmacologically targeting the MPC in short-term human hair follicle *ex vivo* organ culture experiments using UK-5099, we induced metabolic stress-responsive proliferative arrest throughout the human hair follicle epithelium, including within Keratin 15+ eHFSCs. Through transcriptomics, MPC inhibition was shown to promote a gene expression signature indicative of disrupted FGF, IGF, TGFβ and WNT signalling, mitochondrial dysfunction, and activation of the integrated stress response (ISR), which can arrest cell cycle progression. The ISR, mediated by the transcription factor ATF4, is activated by stressors including amino acid deprivation and ER stress, consistent with MPC inhibition within our model. Using RNAScope, we confirmed the upregulation of both *ATF4* and the highly upregulated ATF4-target gene *ADM2* on human hair follicle tissue sections *in situ*. Moreover, treatment with the ISR inhibitor ISRIB attenuated both the upregulation of *ADM2* and the proliferative block imposed via MPC inhibition. Together, this work reveals how the human hair follicle, as a complex and metabolically active human tissue system, can dynamically adapt to metabolic stress.

## Introduction

Hair growth and cycling is a metabolically demanding process [1, 2]. Especially during the anagen phase of human scalp hair follicles, where highly complex hair matrix keratinocyte proliferation and differentiation, controlled by epithelial-mesenchymal interactions, can go uninterrupted for years on end [3, 4].

It has long been thought that human hair growth is supported by aerobic glycolysis [1, 2]. Indeed, previous work has shown that inhibition of murine MPC1, blocking mitochondrial pyruvate uptake, activates telogen epithelial hair follicle stem cells (eHFSCs) through the enhancement of cytosolic lactate generation via lactate dehydrogenase (LDH) [5]. However, targeted induction of mitochondrial defects and TCA cycle dysfunction, which can result from MPC disruption [6], can activate the integrated stress response (ISR) [7–9].

The ISR is triggered by the activity of one of four kinases, namely HRI, GCN2, PERK and PKR, that phosphorylate eIF2α following the detection of cellular stress, such as amino acid deprivation or endoplasmic reticulum stress [7]. The phosphorylation of eIF2α inhibits global protein synthesis while permitting the translation of select genes, including the transcription factor ATF4. ATF4’s target genes can promote either cell survival, or apoptosis (e.g. via CHOP) if cellular adaption processes fail) [7].

To further explore these metabolic and metabolic stress mechanisms in a human system, we experimentally targeted the MPC in anagen VI scalp hair follicles *ex vivo* using the MPC inhibitor UK-5099, and conducted *in situ* cell cycle analyses, followed by transcriptomics and targeted RNA *in situ* hybridization.

Our results indicate that blocking mitochondrial pyruvate uptake via MPC inhibition not only induces cell cycle arrest and mitochondrial dysfunction but also disrupts the expression of key hair follicle signalling network genes and activates the ISR pathway [7] in human anagen hair follicles.

## Materials and methods

### Hair follicle organ culture

Human hair follicle samples were obtained from consenting patients from the Crown Clinic, Manchester, and were handled and stored in line with HTA regulations and ethical approval. Human hair follicles were cultured serum-free in human hair follicle medium comprising Williams E medium (containing 0.025 g/L sodium pyruvate), supplemented with penicillin (100 U/ml), streptomycin (100 μg/ml), insulin (10 μg/ml) hydrocortisone (10 ng/ml)) and l-glutamine (2 mM) [10, 11]. Human hair follicles were treated with UK-5099 (10 and 40 μM Tocris #4186) for 2 days, or 40 μM UK-5099 with and without trans-ISRIB (500 nM, tocris #5284) for 2–3 days before being isolated, embedded in frozen medium and frozen in liquid nitrogen, or stored in RNAlater. Hair follicle samples in RNAlater were extracted using RNeasy Micro Kit (Qiagen #74004). Frozen hair follicles were cryosectioned onto SuperFrost Plus™ slides at a thickness of 7 and 10 μm for immunofluorescence and RNAScope respectively.

### Staining, microscopy and analysis

Frozen human hair follicle tissue sections were fixed in acetone chilled to -20°C, and immunofluorescence was conducted as previously described [10] using antibodies to detect MPC1 [D2L9I] (Cell Signaling #14462) (1:100), PDK1 [2H3AA11] (Abcam #ab110335), Ki-67 [SP6] (Abcam #ab16667) (1:50), Cleaved Caspase-3 (Asp175) (Cell signalling #9661) (1:50), TOMM20 [EPR15581-54] (Abcam #ab186735) (1:100). EdU labelling was conducted as previously described using Click-iT™ EdU Alexa Fluor™ 488 Imaging Kit (Thermo Fisher #C10337) [12]. RNA fluorescent *in situ* hybridisation was performed using RNAscope® Multiplex Fluorescent V2 Assay (ACD # 323270) and probes Hs-ADM2 (ACD #822861) and Hs-ATF4-C2 (ACD #405741-C2).

Image analysis and quantification was performed in ImageJ (National Institutes of Health NIH) using the measure function to obtain brightness values, or the Multi-point tool to quantify FISH signal or the total number of positive cells within a defined reference area. Statistical analysis and data presentation was performed within GraphPad Prism 9 (GraphPad Software).

### RNA sequencing and analysis

Total RNA was submitted to the University of Manchester Genomic Technologies Core Facility (GTCF). Quality and integrity of the RNA samples were assessed using a 4200 TapeStation (Agilent Technologies) and then libraries generated using the Illumina® Stranded mRNA Prep. Ligation kit (Illumina, Inc.) according to the manufacturer’s protocol. Briefly, total RNA (typically 0.025-1ug) was used as input material from which polyadenylated mRNA was purified using poly-T, oligo-attached, magnetic beads. Next, the mRNA was fragmented under elevated temperature and then reverse transcribed into first strand cDNA using random hexamer primers and in the presence of Actinomycin D (thus improving strand specificity whilst mitigating spurious DNA-dependent synthesis). Following removal of the template RNA, second strand cDNA was then synthesized to yield blunt-ended, double-stranded cDNA fragments. Strand specificity was maintained by the incorporation of deoxyuridine triphosphate (dUTP) in place of dTTP to quench the second strand during subsequent amplification. Following a single adenine (A) base addition, adapters with a corresponding, complementary thymine (T) overhang were ligated to the cDNA fragments. Pre-index anchors were then ligated to the ends of the double-stranded cDNA fragments to prepare them for dual indexing. A subsequent PCR amplification step was then used to add the index adapter sequences to create the final cDNA library. The adapter indices enabled the multiplexing of the libraries, which were pooled prior to cluster generation using a cBot instrument. The loaded flow-cell was then paired-end sequenced (76 + 76 cycles, plus indices) on an Illumina HiSeq4000 instrument. Finally, the output data was demultiplexed and BCL-to-Fastq conversion performed using Illumina’s bcl2fastq software, version 2.20.0.422.

Unmapped paired-end sequences from an Illumina HiSeq 4000 sequencer were assessed by FastQC (http://www.bioinformatics.babraham.ac.uk/projects/fastqc/). Sequence adapters were removed, and reads were quality trimmed (to phred score q20) using Trimmomatic_0.36 (PMID: 24695404). The reads were mapped against the reference human (hg38) genome and counts per gene were calculated using annotation from GENCODE 39 (http://www.gencodegenes.org/) using STAR_2.7.7a (PMID: 23104886). Normalisation, Principal Components Analysis and differential expression was calculated in DESeq2_1.30.1 using default settings, with alpha for independent filtering set to 0.05 (PMID:25516281**).** Pathway analysis was performed using Ingenuity Pathway Analysis software (Qiagen) and Reactome Analysis Tool. Analysis parameters: Log2fc -1.0 / 1.0; padj <0.05 = 1206 analysis ready molecules. N = 4 donors (with 5–6 anagen hair follicles per condition, per donor).

## Results and discussion

### MPC1 is expressed throughout the human hair follicle

**Fig 1:**
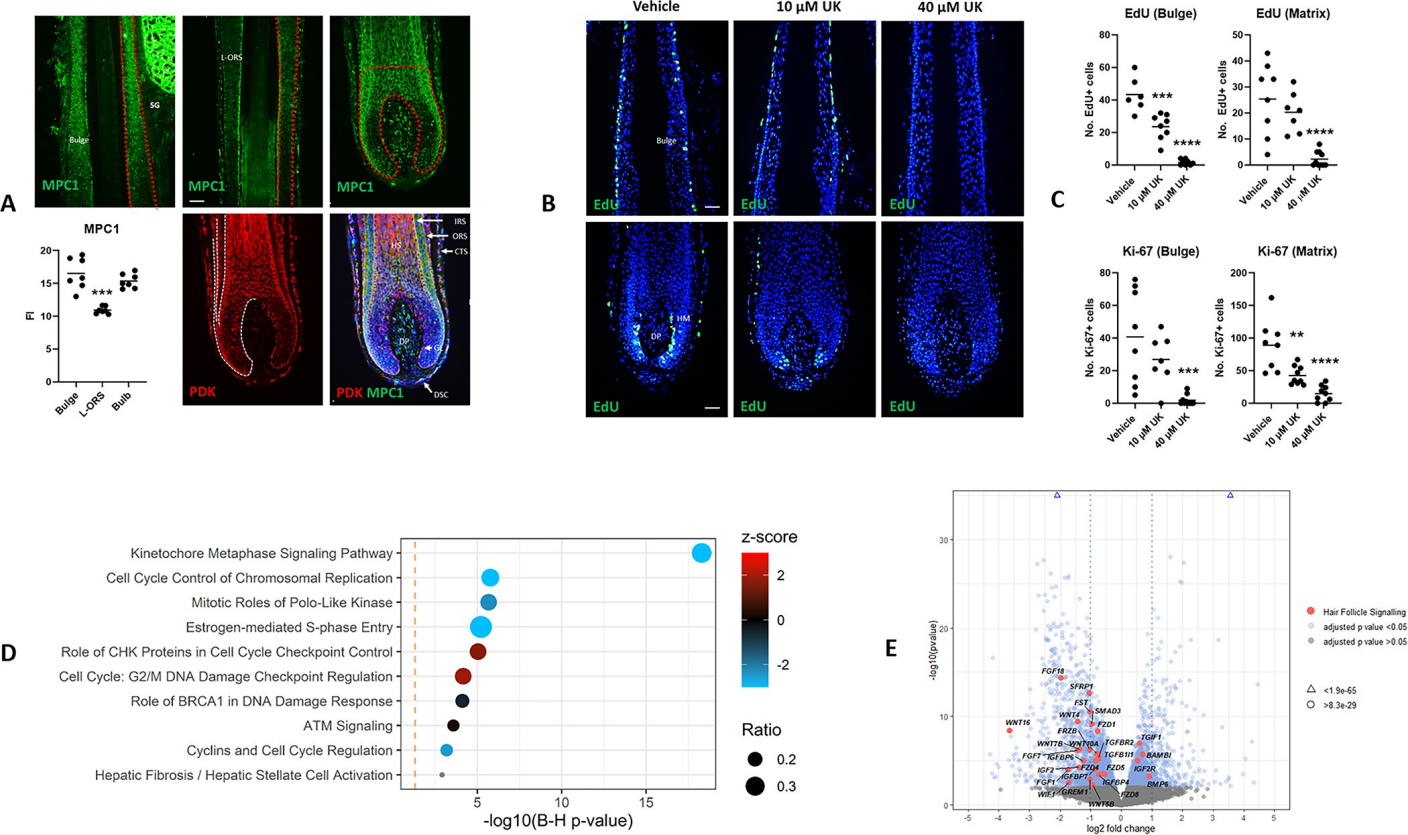
MPC1 is expressed in the human hair follicle and MPC inhibition stalls cell cycle progression and disrupts the expression of hair follicle signalling pathway genes. A) MPC1 and PDK immunoreactivity in the human hair follicle. CTS–connective tissue sheath. DP–dermal papilla; DSC–dermal sheath cup; GL–germinative layer; HS–hair shaft; IRS–inner root sheath; L-ORS–lower outer root sheath; SG–sebaceous gland. Regional analysis (zones of analysis indicated by red dashed lines) performed on 7 anagen hair follicles from 3 donors. Mann Whitney test, p value *** 0.0006. Scale bar 50 μm. B) Fluorescent EdU labelling on human hair follicle tissue sections shows how UK-5099 treatment blocks DNA replication in the hair follicle both within the bulge epithelium and hair matrix (HM). DP—dermal papilla. Scale bar 50 μm. C) Quantitative analysis of EdU and Ki-67 in the bulge and hair matrix following UK-5099 treatment. Ordinary One-way Anova with Multiple Comparisons. EdU analyses: Adjusted p-values *** 0.0002, **** <0.0001. Ki-67 analyses: Adjusted p-values ** 0.0018; *** 0.0006; **** <0.0001. N = 2–3 donors (6–10 independent anagen hair follicles per condition). Plotted line is the mean. D) Dot plot of the top 10 enriched IPA pathways following 40 μM UK-5099 treatment. Analysis conducted on 1206 genes with 2-fold change and padj <0.05. See also S3 Fig in **S1 File**. E) Volcano plot annotated with differentially expressed genes involved with FGF, IGF, TGFβ and Wnt signalling with an adjusted p value < 0.05 following treatment of human hair follicles with 40 μM UK-5099.

We first stained tissue sections from freshly isolated human hair follicles for MPC1, which we determined to be expressed throughout the anagen hair follicle, including within the Keratin 15 (K15)+ human epithelial hair follicle stem cell (eHFSC) bulge compartment [13–15] **(Fig 1A and S1A Fig in S1 File)**. MPC1 was relatively decreased in the lower outer root sheath (L-ORS) versus the bulge eHFSC compartment and the bulb. This supports that aerobic glycolysis occurs in the L-ORS [1, 2]. Dual labelling with Pyruvate Dehydrogenase Kinase (PDK) highlighted where PDK is expressed in the hair follicle relative to MPC1 **(Fig 1A)**, where it may act to restrict pyruvate oxidation favouring the conversion of pyruvate to lactate. As PDK was limited in the layers of inner root sheath (IRS) (presumed Huxley and IRS cuticle layers) and the dermal papilla (DP), these cell types may be more reliant on mitochondrial pyruvate oxidation.

### MPC inhibition promotes cell cycle arrest in proliferating matrix keratinocyte and epithelial progenitor cell populations

Next, we treated human hair follicles within an *ex vivo* 3D tissue culture model [10] with the MPC inhibitor UK-5099 [5], and analysed cell cycle parameters (i.e. Ki-67, EdU incorporation [3, 12]) *in situ* within the bulge eHFSC and hair matrix compartments where immunoreactivity for MPC1 was seen to be relatively abundant.

Analysis of the number of Ki-67+ and EdU+ cells [3, 10, 12] showed a significant decrease in both of these regions, showing that MPC inhibition arrests proliferation in human anagen bulge eHFSCs and hair matrix keratinocytes **(Fig 1B and 1C and S2 Fig in S1 File)**. Such disruption to normal hair follicle keratinocyte proliferation, if sustained, could lead to dystrophic anagen or catagen [16]. It is worth noting that MPC1 protein expression does not localise to just proliferating keratinocytes in the hair follicle **(Fig 1A)**, therefore the anti-proliferative effects of UK-5099 may be exerted indirectly from signals emanating from neighbouring cell populations (e.g. such as the DP).

These findings were unexpected with respect to previous work, whereby targeting the MPC activates murine telogen eHFSCs [5]. However, these observations could depend on hair cycle stage, reflectbiological differences between murine back skin versus human scalp hair follicles (e.g. alongside anatomical, hair cycle length/pattern and eHFSC marker expression differences [13, 14]), or culture conditions (i.e. short term 48h culture in the presence of UK-5099). Nevertheless, our results affirm the importance of mitochondrial pyruvate oxidation in cells of the human hair follicle during *ex vivo* culture. This finding does not exclude the possibility that other compartments of the hair follicle may rely on aerobic glycolysis, which could supply lactate to other regions for oxidation [2].

To confirm cell cycle arrest within the eHFSC compartment, we conducted K15/Ki-67 dual immunofluorescence staining. This showed that UK-5099 treatment blocks K15+ eHFSC proliferation during *ex vivo* human hair follicle organ culture as evidenced by a loss of Ki-67 expression in these cells, but this did not significantly influence K15+ immunoreactivity in the bulge **(S1A Fig in S1 File)**. On the other hand, analysis of the bulge eHFSC marker CD200 [13–15] showed that UK-5099 significantly increased immunoreactivity **(S1B Fig in S1 File)**. Increased CD200 protein expression could represent the maintenance of quiescent eHFSC character in the bulge [13, 15] following MPC inhibition.

### RNA-seq analysis reveals enrichment of cell cycle progression and checkpoint gene pathways in the hair follicle following MPC inhibition

To shed further light on the effects of MPC inhibition on the human hair follicle, we conducted RNA-Seq on hair follicles treated with UK-5099. Using ingenuity pathway analysis (IPA) we found that within the top 10 hits, cell cycle progression pathways were downregulated and cell cycle checkpoints were upregulated (adjusted p value = <0.05; 2-fold increase/decrease, 1206 genes analysed) **(Fig 1D, S3 and S4 Figs in S1 File)**. Together, this shows that inhibition of the MPC promotes cell cycle arrest in the hair follicle by downregulating cell cycle progression genes and upregulating cell cycle checkpoint genes. RNA-seq revealed that *K15* gene expression was unchanged following UK-5099 treatment **(S5A Fig in S1 File)** as seen with protein expression data. *CD200* expression was significantly decreased by UK-5099 (*padj* = 0.0025; 0.62 log2 fold change) **(S5B Fig in S1 File).** This did not correlate with the increased CD200 protein expression observed in the bulge. However, as RNA-seq was performed on whole anagen hair follicles, this may perhaps represent changes in *CD200* gene expression outside of the bulge eHFSC zone (i.e. within non-epithelial cell compartments).

### MPC inhibition disrupts the expression of hair follicle signalling network genes

Next, we screened differentially expressed genes (parameters: adjusted p value = <0.05; 2-fold increase/decrease) for FGF, IGF, TGFβ and WNT pathway members and regulators that influence the biology of the hair follicle.

This showed that MPC inhibition decreases FGF1, FGF7 (KGF) and FGF18 gene expression [17, 18] **(Fig 1E)**. Moreover IGF signalling was also downregulated, with increased expression of IGF-inhibitory IGF2R, and decreased expression of IGF2, IGFBP4, IGFBP6 and IGFBP7 [19, 20] **(Fig 1E)**.

Furthermore, the expression of BAMBI [21] and TGIF1 [22] was increased, while the expression of SMAD3, TGFBR2 and TGFB1I1 [23] expression was decreased **(Fig 1E)**, suggesting that MPC inhibition negatively regulates TGFβ signalling. The expression of the BMP inhibitor FST [24] was also downregulated, while BMP6 was upregulated [25, 26] **(Fig 1E)**. Finally, treatment with UK-5099 treatment significantly decreased the expression of WNT ligands (WNT10A, WNT16, WNT4, WNT5B, WNT7B), receptors (FZD1, FZD4, FZD5, FZD8), and negative regulators of WNT signalling (FRZB, SFRP1, GREM1, and WIF1) [27, 28] **(Fig 1E)**.

Together, these results show that MPC inhibition disrupts key signalling networks in the hair follicle, which may directly contribute to a complete loss of proliferation. However, specific analysis of human hair keratins (KRT31-40; KRT81-86 [29]) showed that only KRT38 expression was significantly altered (*padj* = 0.02; 0.70 log2 fold change) following UK-5099 treatment. This suggests that MPC inhibition and subsequent cell cycle arrest do not immediately affect differentiation (e.g., in the hair shaft and inner root sheath) within our short-term experimental model, and that such effects may only become apparent after several additional days of growth arrest [3].

### MPC inhibition downregulates functionally important mitochondrial and glycolysis genes, and promotes mitochondrial dysfunction in the hair follicle

**Fig 2:**
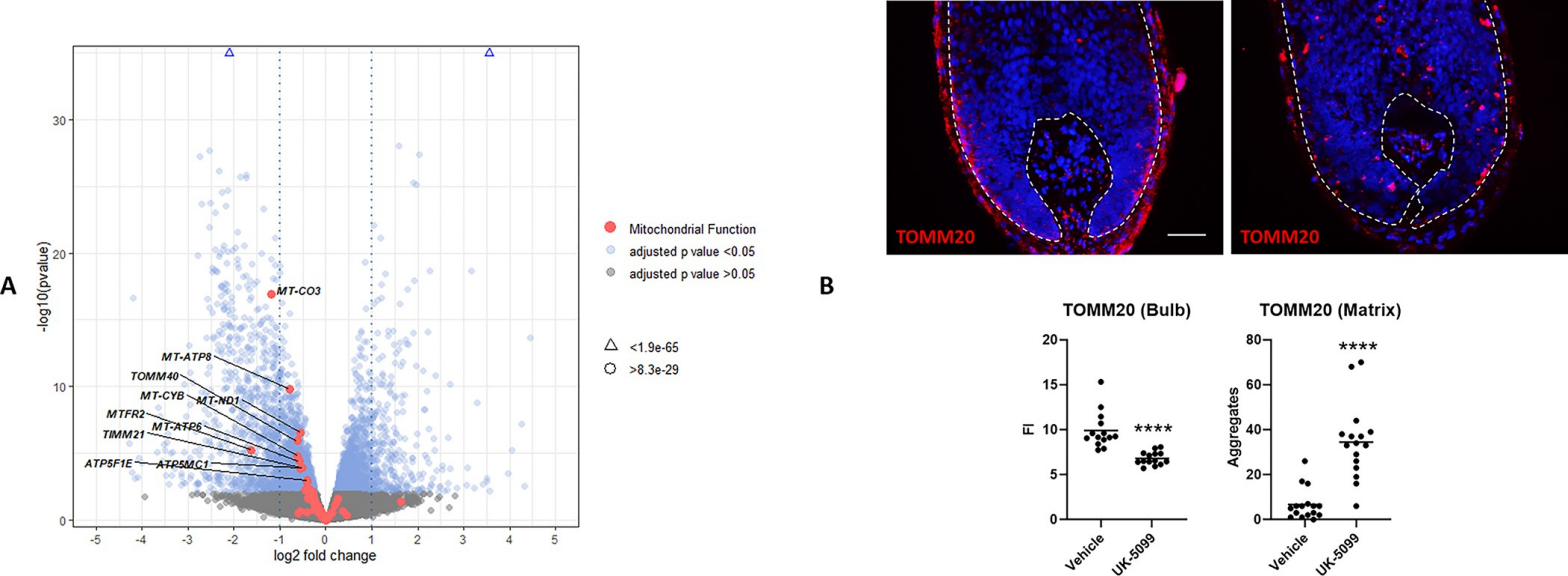
MPC inhibition promotes mitochondrial dysfunction in the human hair follicle. A) Volcano plot annotated with differentially expressed genes involved with mitochondrial function with an adjusted p value < 0.05 following treatment of human hair follicles with 40 μM UK-5099. B) Staining with the mitochondrial marker TOMM20 comparing vehicle and 40 μM UK-5099 treated human hair follicles. Analysis of intensity (FI) and TOMM20+ aggregates reveals changes in the hair matrix following MPC inhibition. Mann-Whitney test. Exact p-values **** <0.0001. N = 5 donors (16 independent anagen hair follicles per condition). Scale bar 50 μm.

Continuing our analysis of differentially expressed genes in human anagen hair follicles following treatment with the MPC inhibitor UK-5099, we screened for genes involved with glycolysis/anaerobic metabolism [30] and mitochondrial function. This included a targeted screening of 97 genes belonging to the gene group “mitochondrial respiratory chain complexes” (HGNC, April 2024). This revealed that MPC inhibition downregulates several genes associated with glycolysis, including LDHA (**S6 Fig in S1 File**), as well as genes involved with mitochondrial function, including MT-CO3, MT-CYB, MTFR2, MD-ND1, MT-ATP6 and MT-ATP8 **(Fig 2A)**.

To directly study mitochondrial dynamics and function in human hair follicle tissue sections following MPC inhibition, we stained for the mitochondrial marker TOMM20 [31]. This revealed altered TOMM20 immunostaining patterns, whereby staining intensity in the bulb decreased, while the number of TOMM20+ aggregates increased **(Fig 2B)**. These changes, coupled with the observed alterations in gene expression related to mitochondrial function, suggest that MPC inhibition induces mitochondrial dysfunction in the hair matrix.

### MPC inhibition activates the integrated stress response in the human hair follicle

**Fig 3:**
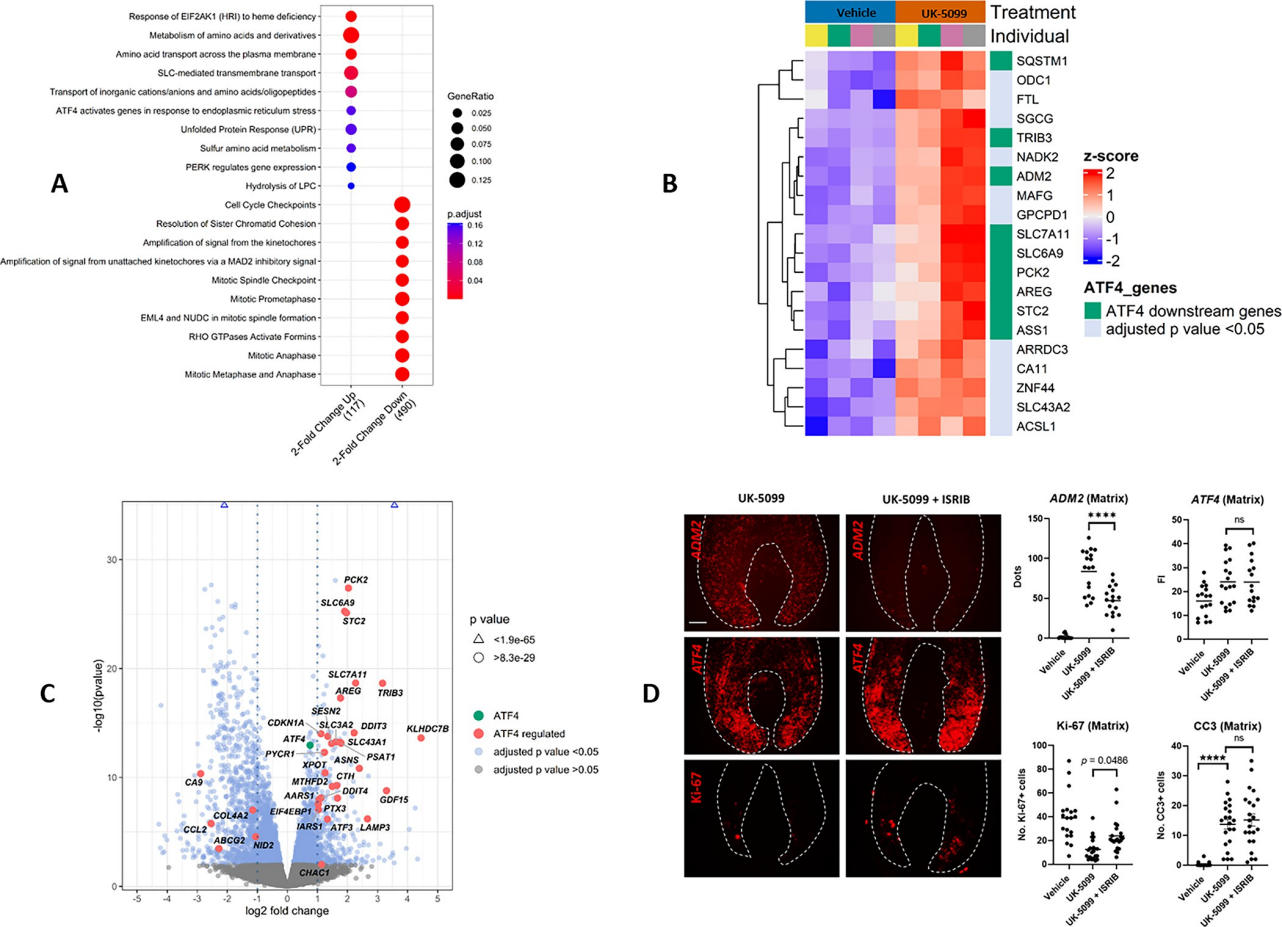
MPC inhibition activates the integrated stress response in the human hair follicle. A) Dot plot of the top enriched pathways for upregulated and downregulated genes, following 40 μM UK-5099 treatment generated using ReactomePA. Analysis was conducted by filtering the top upregulated and downregulated genes (adjusted p value < 0.05 and a 2-fold change), analysing 117 and 490 genes respectively. ReactomePA analysis was performed in R [34]. Results were adjusted for multiple testing using the Benjamini and Hochberg method. B) Heatmap of upregulated genes (from top 100 genes by adjusted p value, see S4 Fig in **S1 File** for heatmap of top differentially expressed genes) in the human hair follicle following 40 μM treatment with the MPC inhibitor UK-5099. Genes reported to be targets of ATF4 are annotated in green. C) Volcano plot with annotated genes identified to be regulated by ATF4 in IPA. 33 annotated ATF4 target genes identified from list of 1206 genes with an adjusted p value < 0.05 and a 2-fold change following treatment of human hair follicles with 40 μM UK-5099. D) Images of *ADM2 and ATF4* (mRNA FISH), and Ki-67 expression in the hair matrix in hair follicles treated with UK-5099 or UK-5099 + ISRIB (ISR inhibitor). Scale bar 50 μm. Quantitative analysis of *ADM2*, *ATF4*, Ki-67 and Cleaved Caspase 3 (CC3) comparing UK-5099 + ISRIB treatment versus UK-5099 alone and/or vehicle (see also S8-S10 Figs in **S1 File**). Ordinary One-way Anova with Multiple Comparisons. Adjusted p-value **** <0.0001. Plotted line is the mean. N = 4–6 donors (16–22 independent anagen hair follicles per condition).

To further examine how MPC inhibition influences the human hair follicle transcriptome, we separately analysed the top downregulated genes and the top upregulated genes separately. We suspected that extensive downregulation of cell cycle-related genes was obscuring other findings within the dataset. Using Reactome pathway analysis (ReactomePA) [32], we confirmed an enrichment of cell cycle-related pathways among the top downregulated genes **(Fig 3A)**. However, pathway analysis of the top upregulated genes revealed enrichment for the pathway “Response of EIF2AK1 (HRI) to heme deficiency” and various metabolic pathways, including amino acid metabolism and transport **(Fig 3A).** HRI is one of four kinases, alongside GCN2, PERK and PKR, known to phosphorylate eIF2α as part of the integrated stress response (ISR) [7]. The ISR can also function to arrest cell cycle progression [33], and the HRI-ISR pathway has been described to induce mitophagy in response to mitochondrial dysfunction [31]. This may relate to changes seen in TOMM20 immunoreactivity in the hair bulb as described above **(Fig 2B)**.

Supporting ISR activation in the hair follicle, several of the top upregulated genes are known targets of the critical ISR transcription factor ATF4, including *ADM2* [35], *PCK2* [36], *SLC6A9* [37], *STC2* [38], *SLC7A11* [39], *TRIB3* [40], *ASS1* [41], *AREG* [42] and *SQSTM1* [43] **(Fig 3B and S4 Fig in S1 File)**. Additionally, our data revealed that ATF4 target genes such as DDIT3 (CHOP) [44], CDKN1A [33], NUPR1 [45], KLHDC7B [46], PSAT1 [47], and SESN2 [48] were significantly upregulated following UK-5099 treatment in the hair follicle **(S7 Fig in S1 File)**. IPA software also identified 33 ATF4 regulated-genes in our dataset **(Fig 3C)**. However, despite predictions by IPA, the genes *NID2*, *ABCG2*, *CCL2* and *CA9* were downregulated by UK-5099 treatment **(Fig 3C)**, contrary to their expected upregulation by ATF4. Notably, *ATF4* itself was significantly upregulated by UK-5099 (padj = 1.19E-11; 0.757 log2 fold change) **(Fig 3C)** [49]. Furthermore, the ATF4 targets NUPR1 and CDKN1A were also identified in our data by IPA as predicted activated upstream regulators.

### RNAScope reveals that MPC inhibition increases the expression of *ADM2* and *ATF4* transcripts in the hair follicle matrix and bulge

To further investigate these findings *in situ* on human hair follicle tissue sections, we employed RNAScope to label *ATF4*, and *ADM2* (an ATF4 target gene which showed a highly significant and high fold change increase; padj = 1.87E-61; 3.56 log2 fold change) in hair follicles treated with UK-5099. Analysis revealed that both *ADM2* and *ATF4* expression were significantly increased by 40 μM UK-5099 treatment in the hair matrix **(S8 Fig in S1 File)**. Additionally, this treatment elevated *ADM2* expression in the bulge and led to a trending, albeit non-significant (*p =* 0.0578*)*, increase in ATF4 expression in the same compartment **(S8 Fig in S1 File)**. Together these data provide evidence that MPC inhibition enhances the expression of genes associated with the ISR pathway in the hair follicle.

### Modulation of the integrated stress response in human hair follicles via ISRIB

Next, we co-cultured human hair follicles with both UK-5099 and the ISR inhibitor ISRIB (500 nM), [50, 51]. This co-treatment significantly decreased *ADM2* expression versus UK-5099 alone within the matrix, and also led to a marginally significant decrease (*p* = 0.0516*)* within the bulge **(Fig 3D and S9A Fig in S1 File),** consistent with previous findings [35]. However, *ADM2* levels remained markedly elevated in the UK-5099 + ISRIB treatment group compared to the control group, suggesting that ISR activation is only partially mitigated by 500 nM ISRIB treatment in our experimental conditions. Furthermore, *in situ ATF4* transcript levels, which increased following UK-5099 treatment, were not significantly affected by ISRIB treatment in either the matrix or bulge, indicating that *ATF4* may be regulated upstream of the ISR following MPC inhibition **(Fig 3D and S9B Fig in S1 File)**. Additionally, the effect of UK-5099 on the number of Ki-67+ cells was partially reversed by ISRIB treatment (*p =* 0.0486) in the matrix and bulge **(Fig 3D and S9C Fig in S1 File).**

Overall, the attenuation of increased *ADM2* expression and the partial restoration of Ki-67 via ISRIB treatment further supports the notion that UK-5099 induces ISR activation in the hair follicle. Notably, UK-5099 treatment alone significantly increased the number of Cleaved Caspase 3+ cells in the hair matrix (but not in the bulge compartment) **(Fig 3D and S10 Fig in S1 File)**, an effect that was unchanged by ISRIB co-treatment. These findings demonstrate that MPC inhibition can lead to apoptosis in the hair matrix, which can occur when the adaptive, pro-survival response arm of the ISR is unsuccessful [7].

### A model for metabolic rewiring in human hair follicles in response to cellular stress

Previous work has shown that MPC deletion or disruption diminishes NADPH production via the TCA cycle, which in turn promotes compensatory glutamine oxidation to sustain NADPH levels, directing glutamine away from glutathione (GSH) synthesis [6, 52, 53]. Additionally, NADPH is essential as a cofactor in converting oxidised glutathione (GSSG) via glutathione reductase [52, 54]. Furthermore, disruption of the TCA cycle and mitochondrial dysfunction have been reported to activate the ISR through impaired redox and amino acid homeostasis [8, 9]. We propose that MPC inhibition similarly disrupts mitochondrial function and cellular metabolism, affecting amino acid availability and redox homeostasis in the hair follicle, thereby promoting ISR activation. Supporting this model, we observed increased expression of the transporters *SLC7A11* and *SLC6A9* which encode xCT and Glyt1, respectively **(Fig 3C)**. These transporters allow uptake of cysteine and glycine respectively, supporting glutathione synthesis [55], and are regulated by ATF4 [37, 39]. Additionally, the ATF4 target *MTHFD2* [56], which contributes to NADPH generation via one-carbon metabolism [57] was upregulated following UK-5099 treatment **(Fig 3C)**. MPC inhibition also increased the expression of *NADK2* **(Fig 3C)** which encodes an enzyme that converts NAD(H) to NADP(H) [58], and *SLC43A2* **(Fig 3C)**, which may facilitatemethionine uptake in hair follicles, which in turn could generate S-adenosyl-L-methionine to support glutathione synthesis [59, 60].

### Differential expression of stress-responsive genes in hair follicles following MPC inhibition

Several stress-responsive genes were differentially expressed in response to MPC inhibition, warranting further investigation in the human hair follicle. Notably, *ADM2*, despite being the most significantly upregulated gene following MPC inhibition, has an unclear role in hair follicle biology. Additionally, *AREG* and *STC2* were also upregulated in response to MPC inhibition. The protein products of these genes could collectively have important physiological effects [61–63] on the hair follicle as stress-responsive secretory factors. Furthermore, our study identified other potentially important genes for hair growth and immune regulation. For instance, *KRT79* [64, 65] was increased, while the immune checkpoint gene *VTCN1* (encoding B7H4) [66–68] showed decreased expression following UK-5099 treatment **(S7 and S11 Figs in S1 File)**.

## Conclusions

Targeting the MPC in the human hair follicle to disrupt mitochondrial pyruvate oxidation activates the ISR and ATF4. This then promotes the downstream expression of genes to promote metabolic rewiring and cell cycle arrest to enable cellular adaption to metabolic stress. This work shows how the human hair follicle, as a highly complex mini-organ, is capable of sensing and dynamically adapting to changes in nutrient availability and redox homeostasis.

By developing on this work, further studying the ISR in the human hair follicle could potentially elucidate novel insights into human hair growth, hair loss disorders, tissue metabolism and ageing [69]. For example, as we show that the activation of the ISR is linked to a proliferative block in the human hair follicle, we hypothesise that the ISR could be aberrantly activated in hair disorders to disrupt normal hair follicle cycling by promoting dystrophic anagen,or catagen. As such, targeting this pathway to mitigate ISR activation (e.g. using ISRIB) could be used to help maintain hair follicles in anagen. However, further work is needed to test the feasibility of blocking ISR activation in the hair follicle, especially since our ability to achieve this using ISRIB was limited.

As mitochondrial dysfunction has been described in scarring alopecia [70], the relationship between this observation and the ISR pathway could be explored. On the other hand, given that we found UK-5099 to enhance the protein expression of CD200 in bulge eHFSCs, hormetic ISR stimulation may be useful in preventing immune privilege collapse in this disorder (bulge immune privilege is supported by CD200 expression, which is decreased in scarring alopecia [13, 71, 72]) Lastly, as ISR activation blocks proliferation, pharmacologically activating the ISR could be protective against chemotherapy-induced damage in the hair follicle via cell cycle-dependent mechanisms [10, 73].

## Supporting information

S1 File (ZIP)
