# Supplementary material for: Activation of the integrated stress response in human hair follicles
Source: PLoS One. 2024 Jun 20;19(6):e0303742. doi: 10.1371/journal.pone.0303742 (PMC11189182; doi:10.1371/journal.pone.0303742)
Supplement: S1 File — (ZIP) [file pone.0303742.s001.zip › S1-11 Fig Captions_corrected.docx]

**S1-11 Captions**

**S1 Fig. Epithelial Stem Cell Markers in Human Hair Follicles Treated with UK-5099.** A) Keratin 15 (K15) and MPC1 dual immunofluorescence highlighting MPC1 expression in the bulge, including within K15+ bulge epithelial stem cells (left panel). K15 and Ki-67 immunofluorescence and analysis demonstrating maintained K15 expression, alongside a loss of Ki-67 expression, in human bulge epithelial stem cells following MPC inhibition (centre and right panels). K15 and CD200 staining conducted as described previously [10–12]. B) CD200 immunofluorescence and analysis in the bulge stem cell compartment. CD200 immunoreactivity in the bulge was significantly increased by 40 µM UK-5099 treatment. Ordinary One-way Anova with Multiple Comparisons: Adjusted p-value **** <0.0001. N = 2-3 donors (14-21 independent anagen hair follicles per condition). Scale bar 50 µm.

**S2 Fig.** **Ki-67 staining on human hair follicle tissue sections following UK-5099 treatment.** Proliferation is blocked in both the bulge epithelium and hair matrix (HM). DP - dermal papilla. Scale bar 50 µm.

**S3 Fig.** **Enriched IPA pathways following 40 µM UK-5099 treatment.** Analysis conducted on 1206 genes with 2-fold change and padj <0.05 N= 4 (5-6 anagen hair follicles per condition, per donor). Results were adjusted for multiple testing using the Benjamini and Hochberg method.

**S4 Fig**. **Heatmap of top 100 differentially expressed genes in the human hair follicle following 40 µM UK-5099 treatment.**

**S5 Fig. Analysis of *K15* and *CD200* gene expression in human hair follicles treated with UK-5099.** A) *K15* normalised read counts were not significantly affected by UK-5099 treatment. B) *CD200* expression is significantly decreased by 40 µM UK-5099 treatment. Data obtained via RNASeq.

**S6 Fig. Volcano plot with annotated glycolysis/anaerobic metabolism genes with an adjusted p value < 0.05 following treatment of human hair follicles with 40 µM UK-5099.**

**S7 Fig.** **Volcano plot annotated with genes of interest in human hair follicles following treatment with 40 µM UK-5099.**

**S8 Fig. MPC inhibition increases *ADM2* and *ATF4* expression *in situ* in human hair follicles.** A) *ADM2* (mRNA FISH) is increased in the hair matrix and bulge following 40 µM UK-5099 treatment. Ordinary One-way Anova with Multiple Comparisons. Adjusted p-values **** <0.0001. N = 2 (5-8 independent anagen hair follicles per condition). Scale bar 50 µm. B) *ATF4* (mRNA FISH) is increased in the hair matrix, and there is a trending increase in *ATF4* in the bulge following 40 µM UK-5099 treatment. Adjusted p-value *** 0.009. N = 2 (4-7 independent anagen hair follicles per condition).

**S9 Fig. Quantitative analysis of *ADM2* and *ATF4* (mRNA FISH), and Ki-67 expression in the bulge region in hair follicles treated with UK-5099 versus UK-5099 + ISRIB.** Images and quantitative analysis of *ADM2* (A), *ATF4* (B) and Ki-67 (C) comparing UK-5099 + ISRIB treatment versus UK-5099 in the bulge. Ordinary One-way Anova with Multiple Comparisons; Unpaired t-test (Ki-67 UK-5099 versus UK-5099 + ISRIB graph only). N = 3-5 (14-20 independent anagen hair follicles per condition). Scale bar 50 µm.

**S10 Fig. Cleaved caspase 3 (CC3) immunoreactivity in hair follicles treated with UK-5099 and UK-5099 + ISRIB.** A) Representative images highlighting increased CC3 in the hair matrix of both UK-5099 and UK-5099 + ISRIB treated hair follicles compared to vehicle. B) Representative images and analysis of the number of CC3+ cells in the bulge comparing vehicle, UK-5099 and UK-5099 + ISRIB treatments. Ordinary One-way Anova with Multiple Comparisons. N = 5 donors (16-18 independent anagen hair follicles per condition). Scale bar 50 µm.

**S11 Fig. Keratin 79, B7H4 and *VTCN1* fluorescence in the human anagen hair follicle.** Keratin 79 (KRT79) (Abcam ab 254953) (1:100) and B7H4 (*VTCN1)* (Abcam ab209242) (1:20) staining in the bulb of untreated human anagen hair follicles. *KRT79* and *VTCN1* are respectively increased and decreased on a transcript level following MPC inhibition (Figure S7). *VTCN1* (ACD Hs-VTCN1 #418081) transcript was labelled within the inner root sheath (IRS) and lower outer root sheath (L-ORS).
