# Supplementary figures and images for: Activation of the integrated stress response in human hair follicles
Source: PLoS One. 2024 Jun 20;19(6):e0303742. doi: 10.1371/journal.pone.0303742 (PMC11189182; doi:10.1371/journal.pone.0303742)

# S1

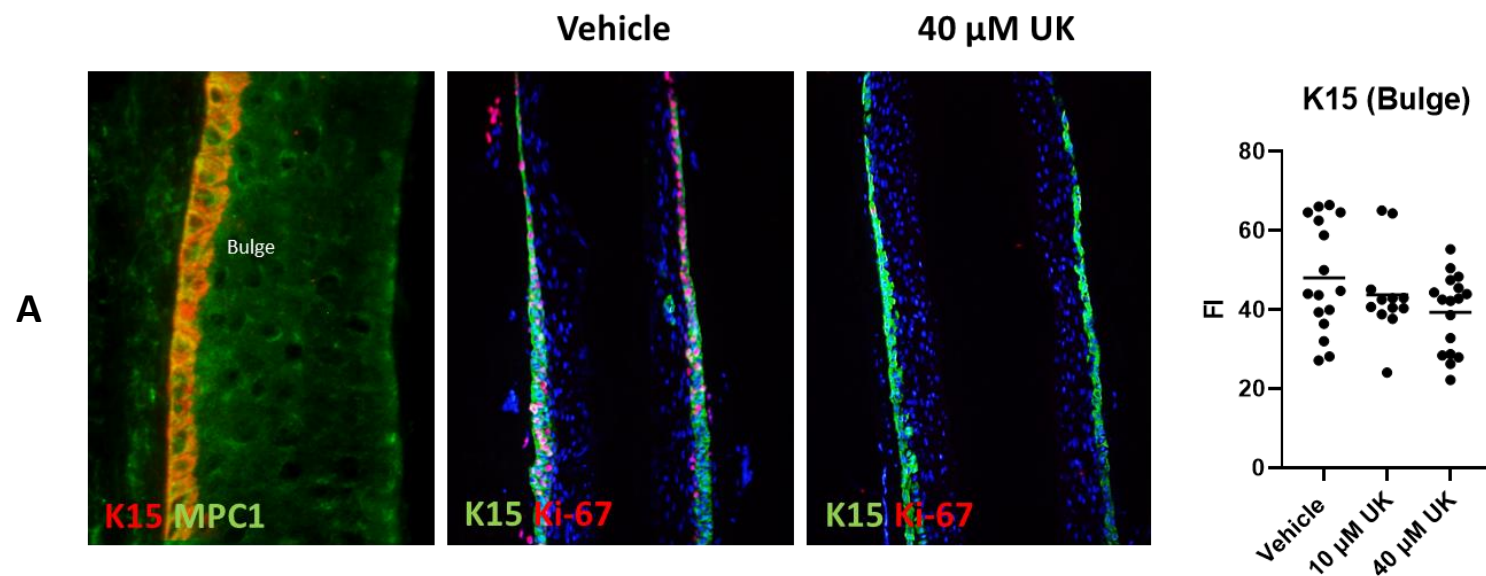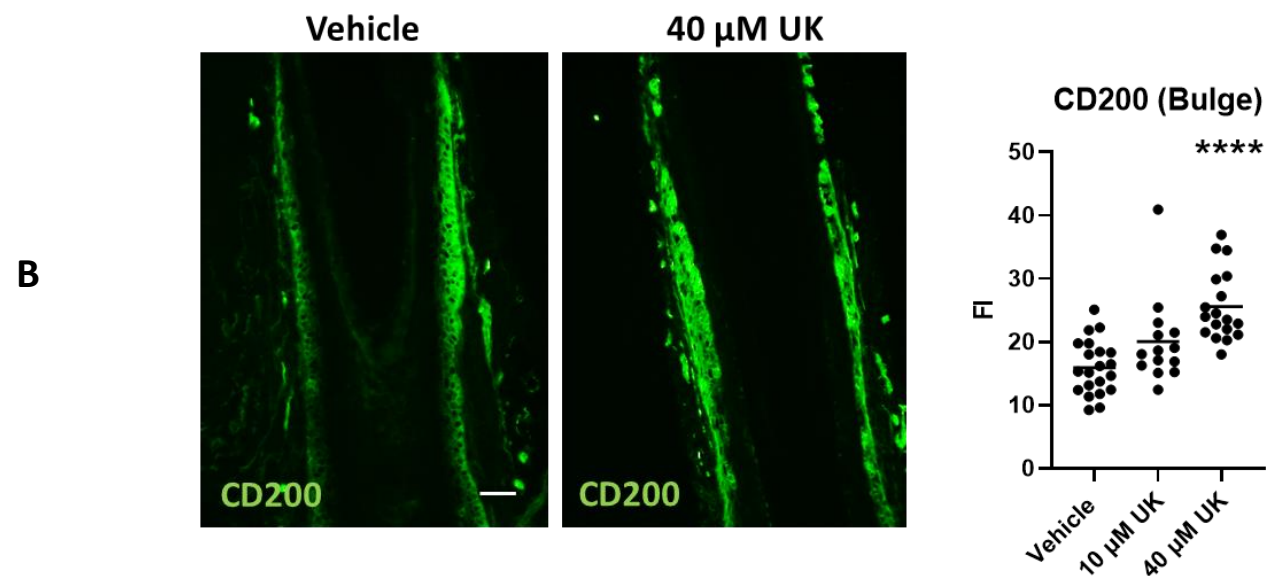

S2

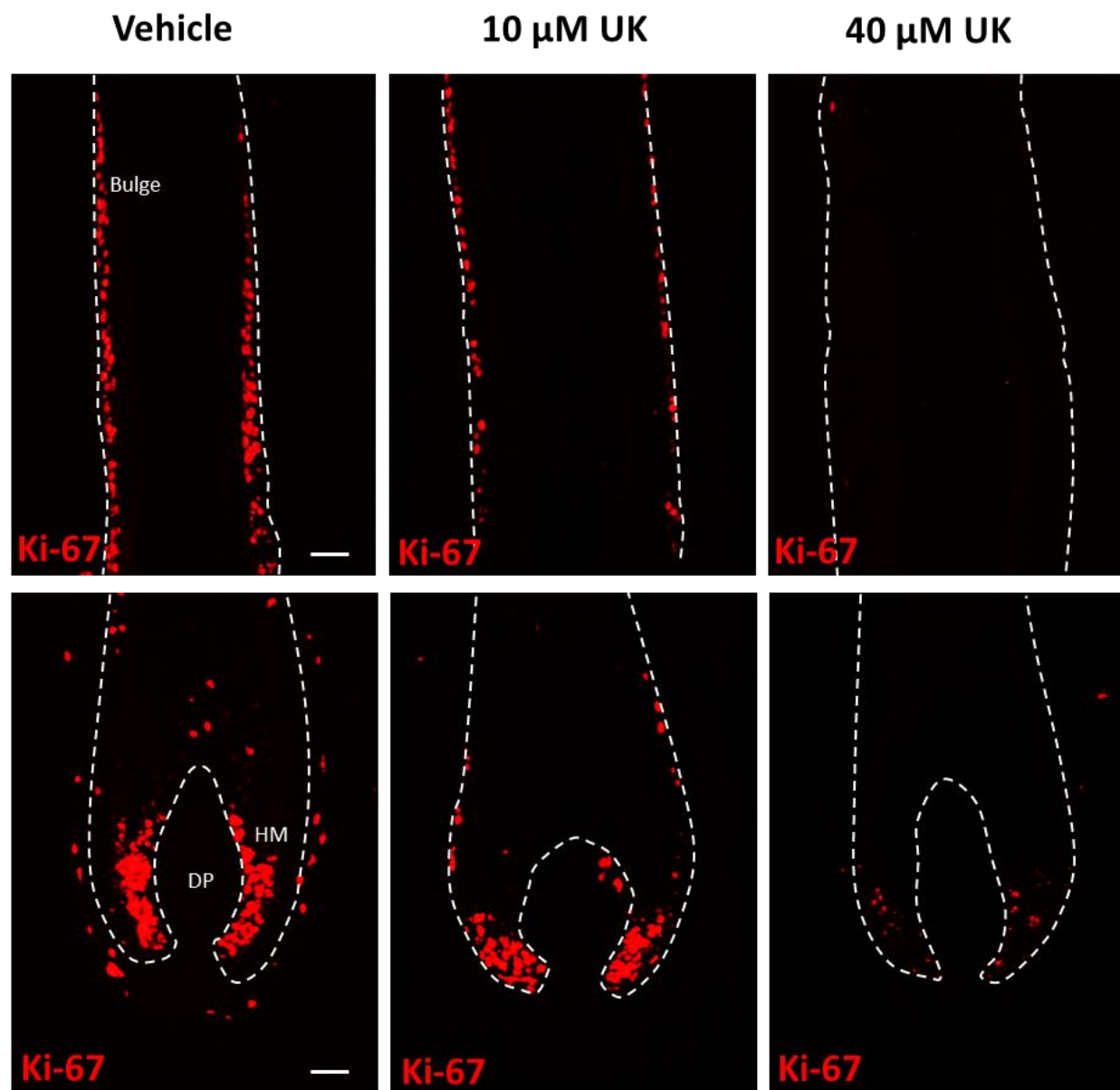

S3

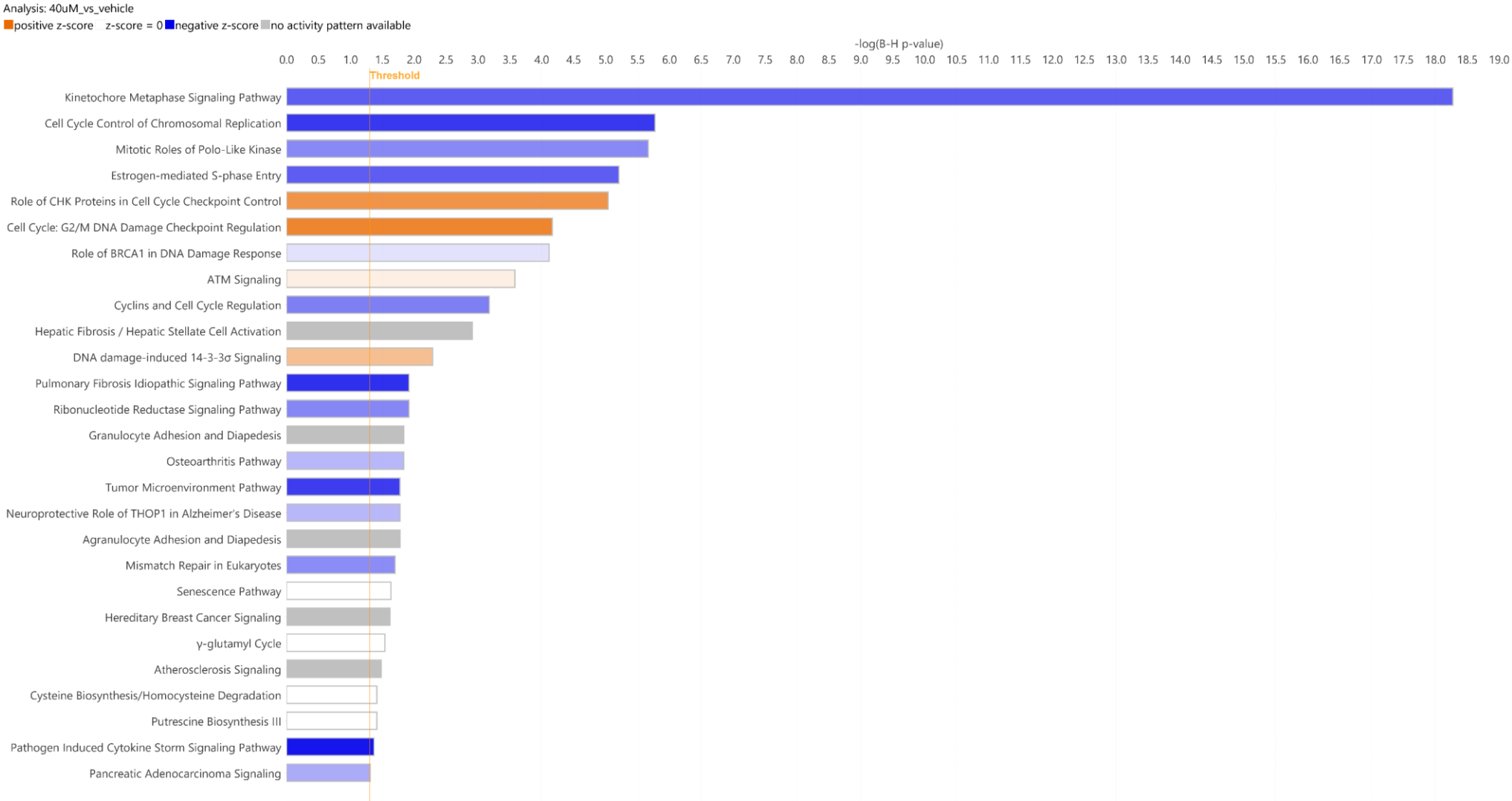

S4

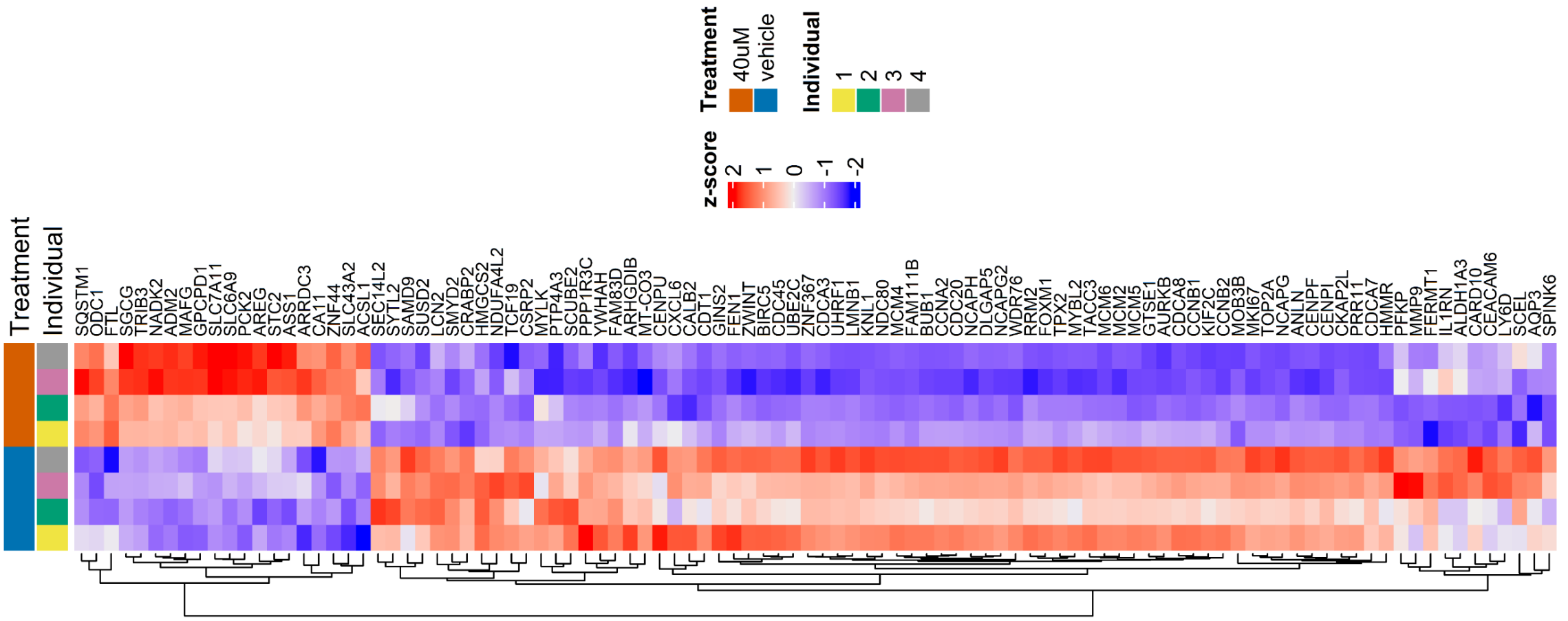

S5

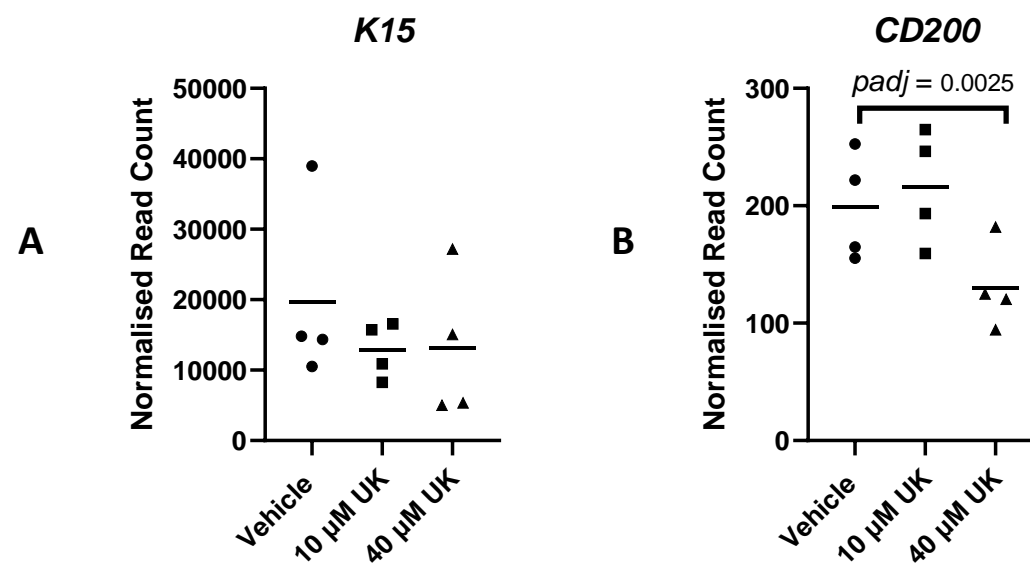

S6

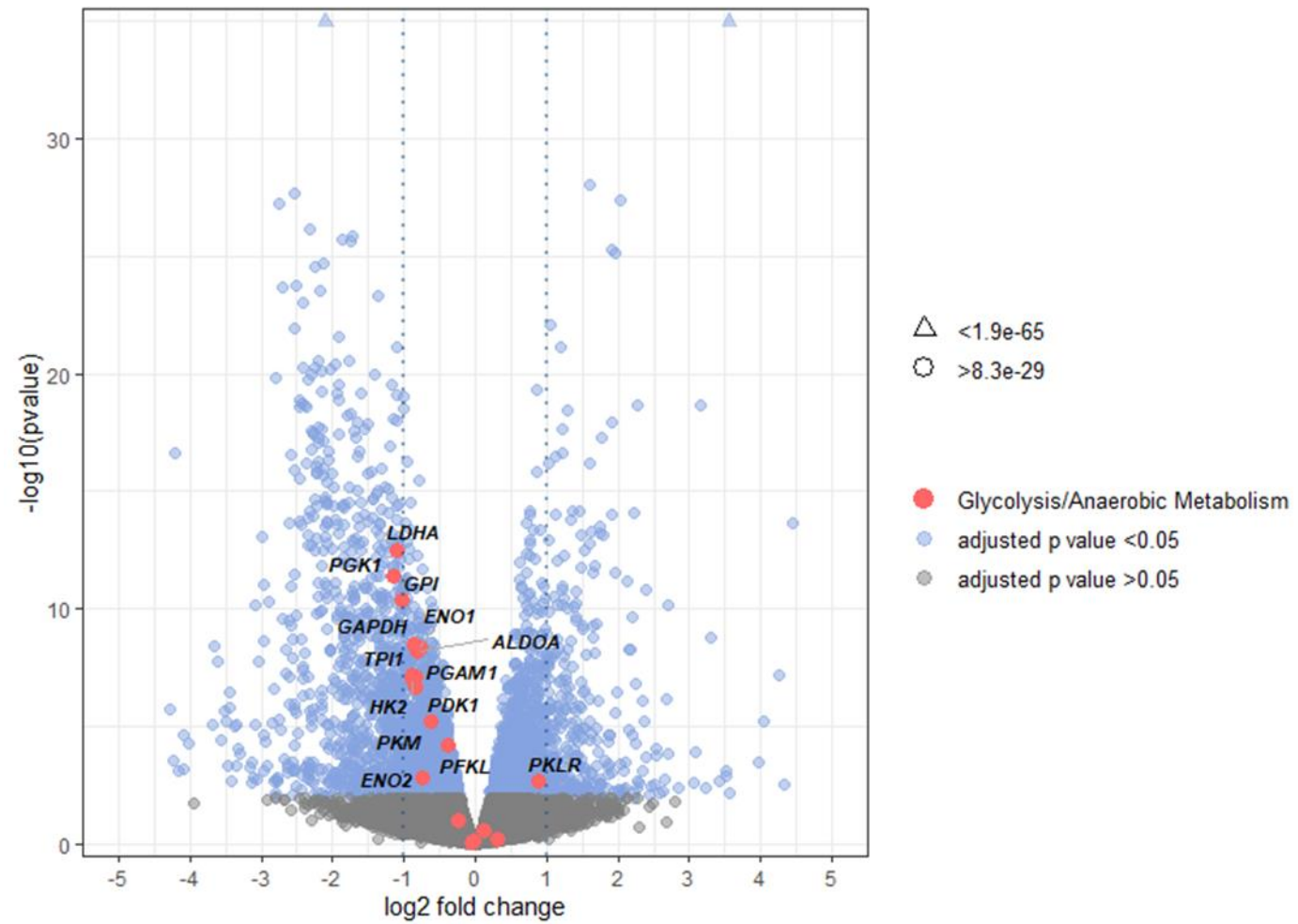

S7

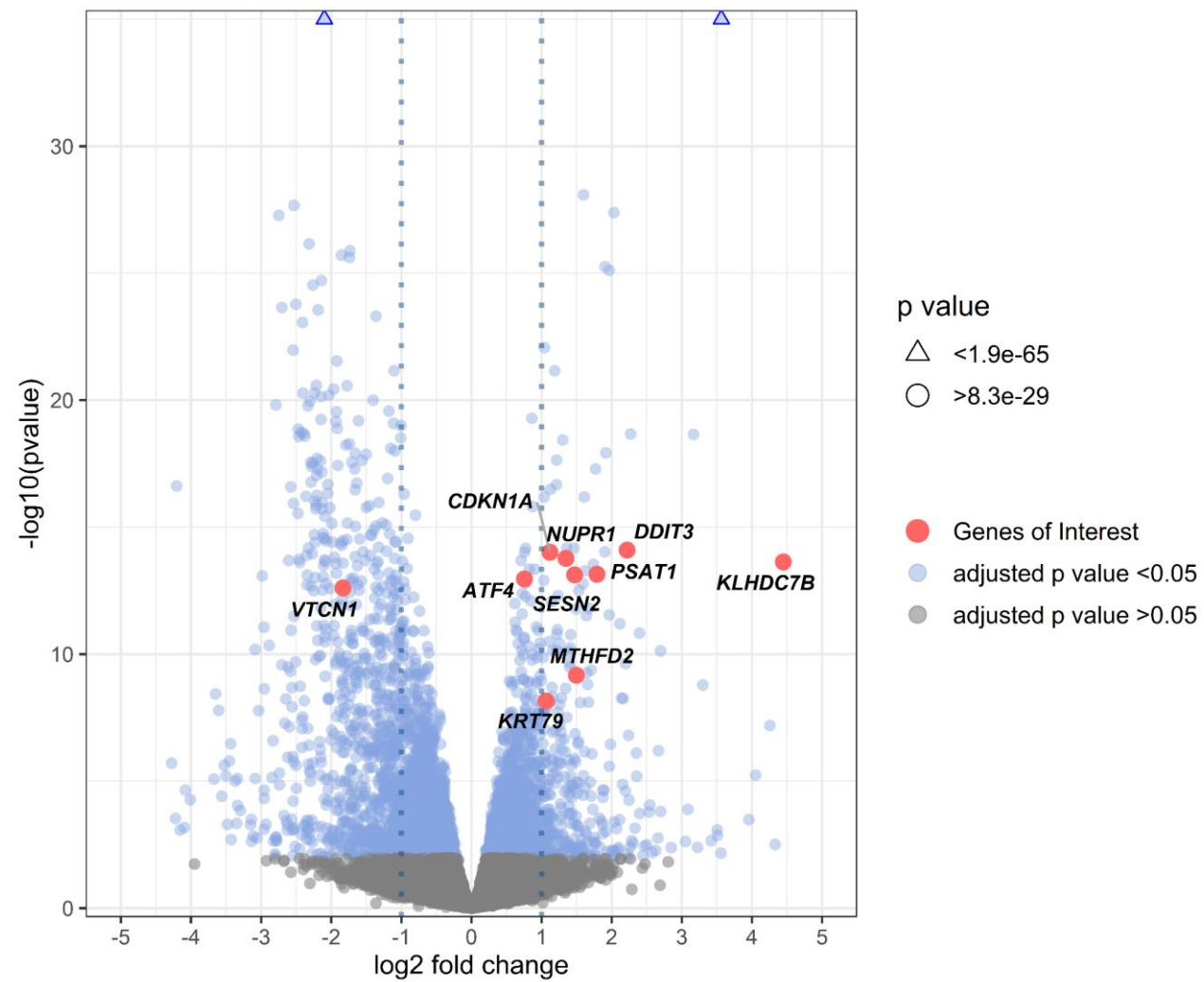

S8

A

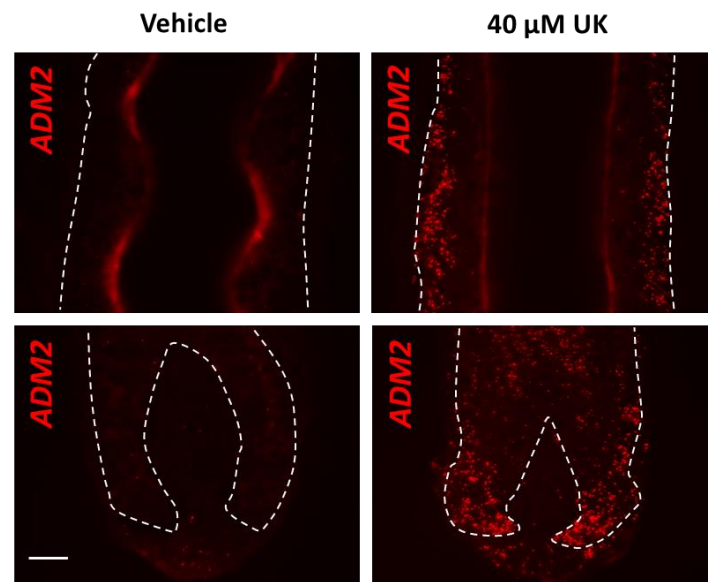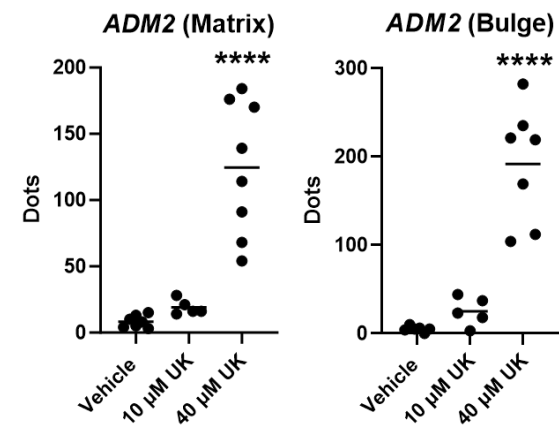

B

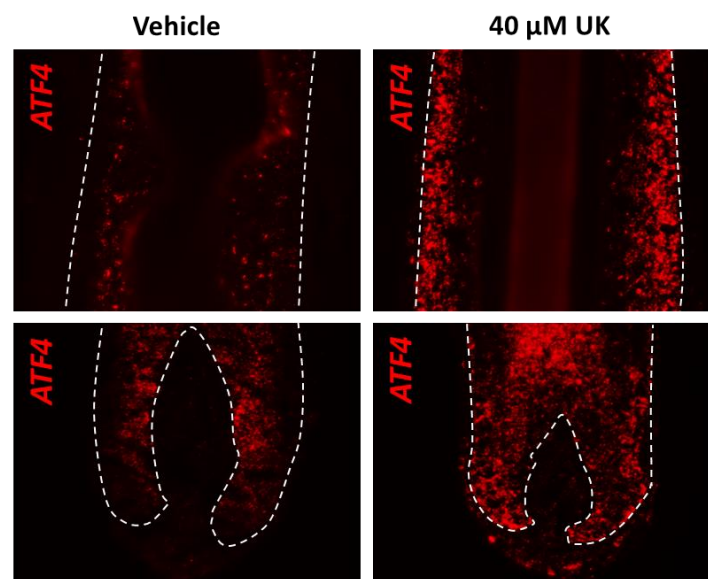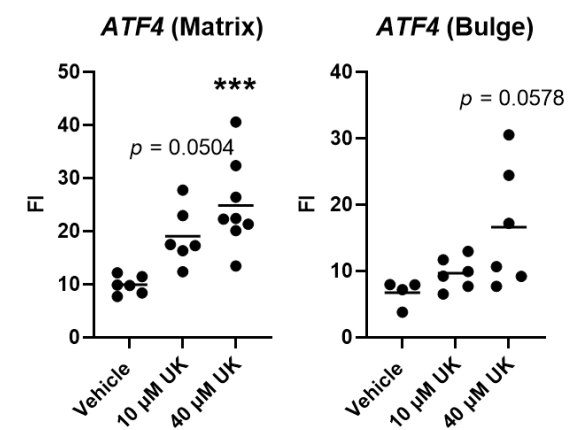

# S9

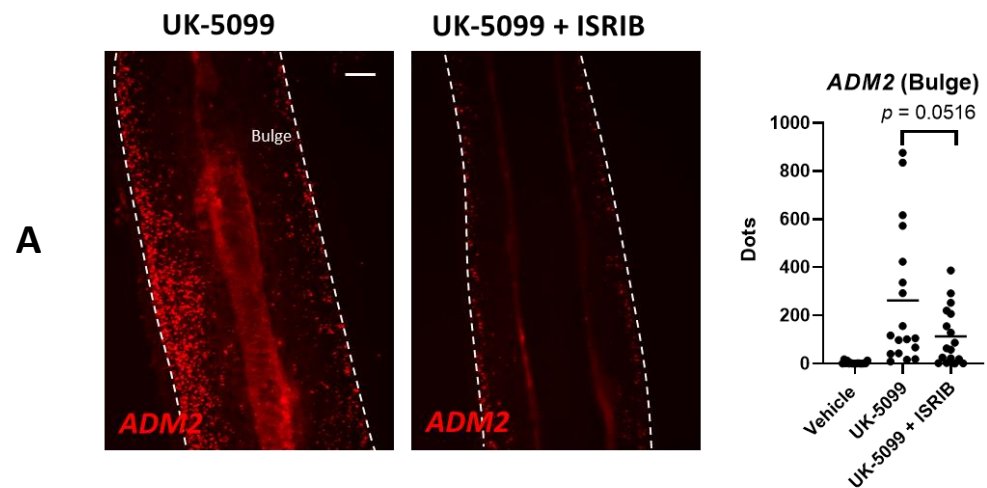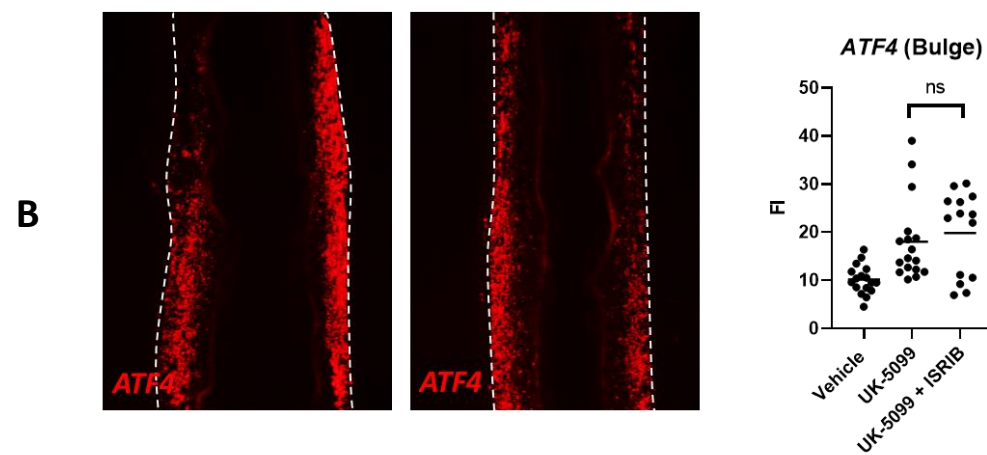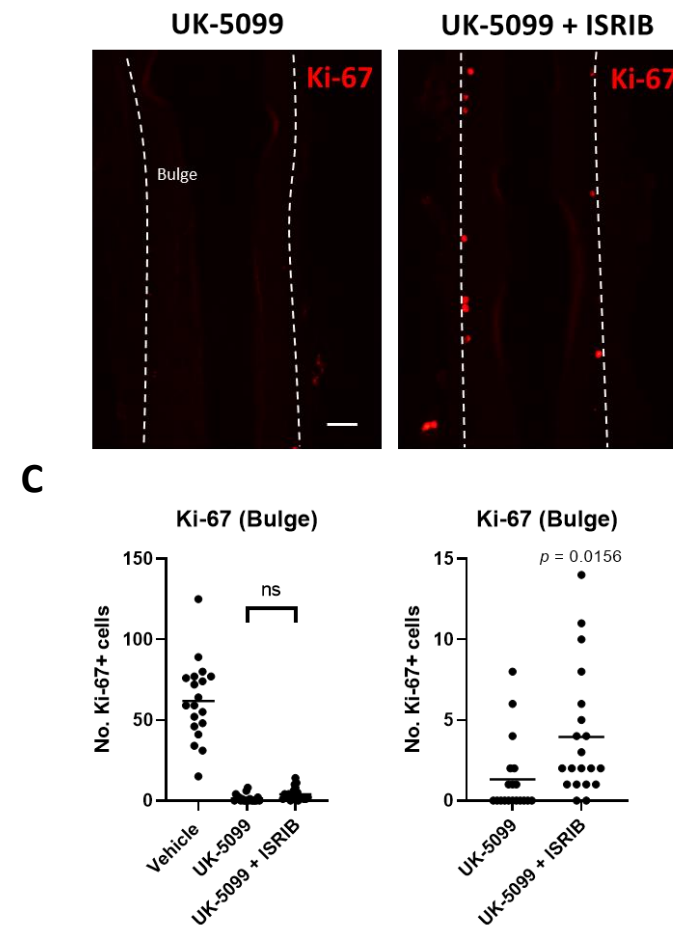

# S10

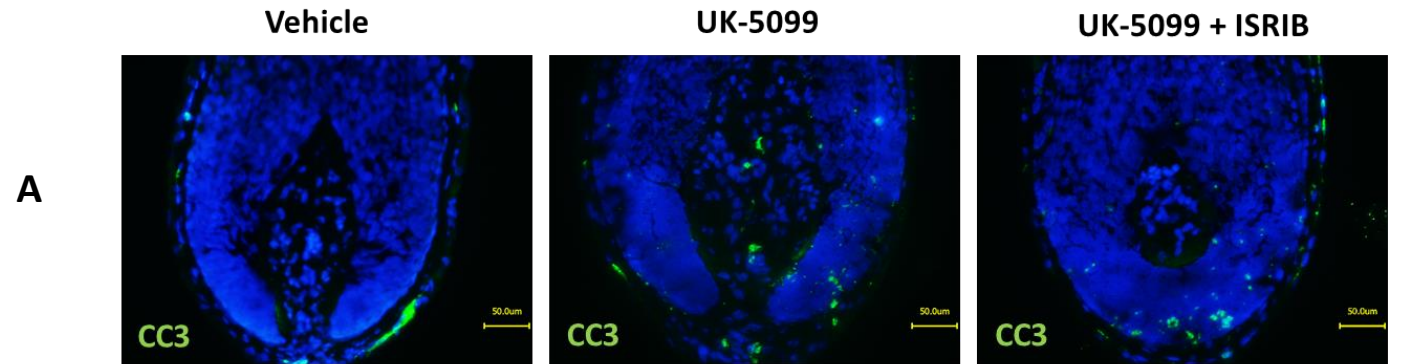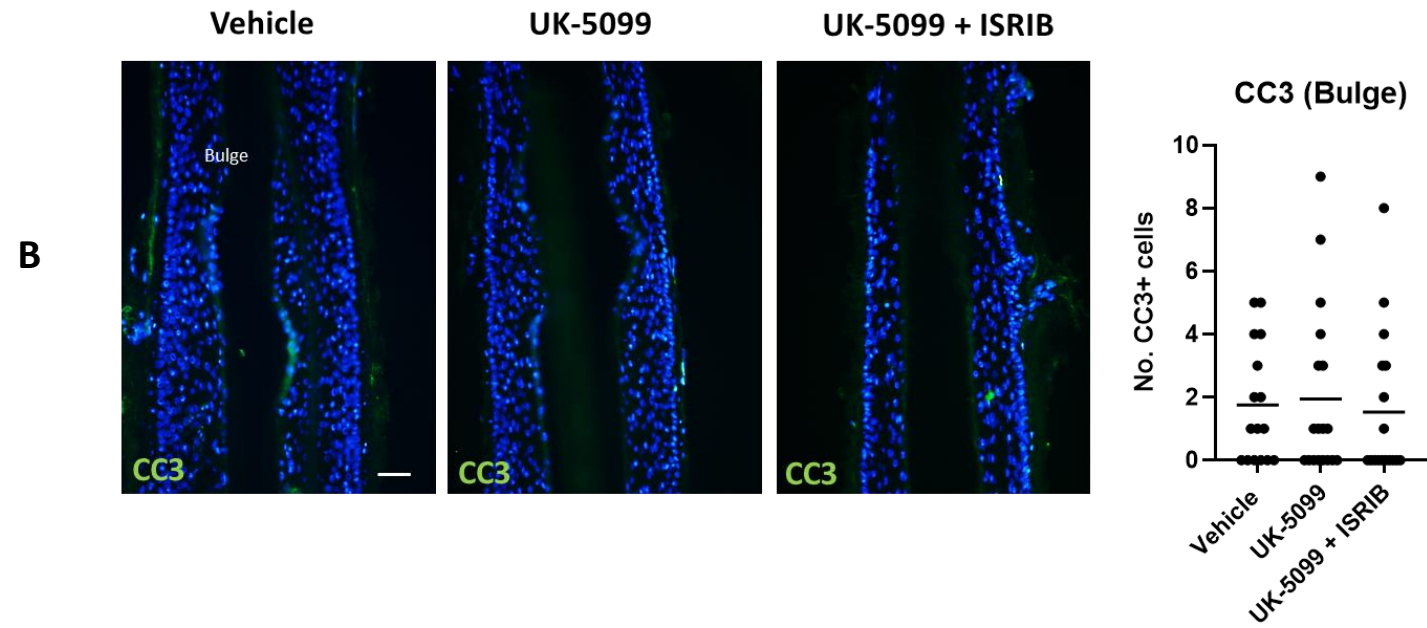

S11

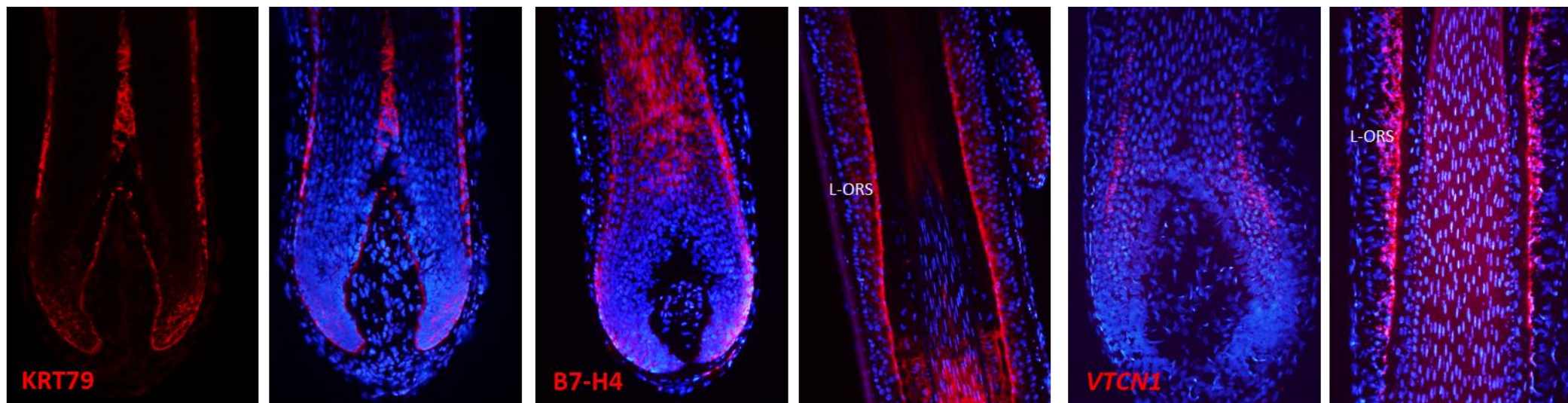

Supplement: S1 File — (ZIP) [file pone.0303742.s001.zip › S1-11 Fig.pdf]
